# Characterisation of the complete mitochondrial genome of *Lucanus chengyuani* (Coleoptera: Lucanidae)

**DOI:** 10.1080/23802359.2019.1674703

**Published:** 2019-10-12

**Authors:** Liang-Jong Wang, Jen-Pan Huang, Shiuh-Feng Shiao, Hsin-Ping Ko, Chia-Hsuan Sung

**Affiliations:** a Division of Forest Protection, Taiwan Forestry Research Institute, Taipei, Taiwan;; b Biodiversity Research Center, Academia Sinica, Taipei, Taiwan;; c Department of Entomology, National Taiwan University, Taipei, Taiwan;; d Taiwan Insect Museum, Taipei, Taiwan;; e Planning and Information Division, Fisheries Research Institute, Keelung, Taiwan

**Keywords:** Mitochondrial genome, *Lucanus chengyuani*, Lucanidae, next-generation sequencing

## Abstract

We sequenced and assembled the complete mitochondrial genome of *Lucanus chengyuani*, from the Alishan, Chiayi County, Taiwan. The length of the complete mitogenome of *L. chengyuani* is 16,926 bp and the mitogenome contains 13 protein-coding, 22 tRNA, and 2 rDNA genes. Nucleotide compositions of the whole mitogenome of *L. chengyuani* are 38.37% for A, 27.96% for T, 23.03% for C, and 10.637% for G. The AT and GC skewness of mitogenome sequence are 0.157 and -0.368, showing the A-skew and C-skew. The reconstructed phylogenetic relationships of 9 Lucanidae species based on 13 mitochondrial protein-coding genes are highly supported. The clade including *Neolucanus maximus* and *Odontolabis cuvera* is sister to the rest of the stag beetle clades, which contains *L. chengyuani* and *L. mazama*. Mitogenomic data from this study will provide useful information for further studies for the population genetics, speciation, biogeography, and conservation of *L. chengyuani* in the future.

Stag beetles are popular insects and draw much attention from stag beetle enthusiasts and taxonomists because of their charismatic male mandibles. The Lucanidae is a diverse family distributed worldwide with about 110 genera and about 1400 described species (Holloway 2007; Fujita 2010). *Lucanus chengyuani* was recently described endemic species to Taiwan (Wang and Ko 2018). It is the third species that has a diurnal mate-searching flight behaviour in the genus *Lucanus* in Taiwan. The habitat of *L. chengyuani* is the open area along forest edges with miscellaneous trees and herbaceous plants. The small body size of *L. chengyuani* likely represents an evolutionary consequence of feeding on low-nutritional food in the habitat (Wang and Ko 2018). This is the first report of a complete mitochondrial genome sequence for the species *Lucanus chengyuani*.

In this study, a single specimen (paraype no. Lcy009) of *L. chengyuani* was collected in Alishan, Chiayi County, Taiwan in May 2018. Total genomic DNA was extracted from the thorax using the QuickExtract™ DNA Extraction Solution kit (Epicentre, Madison, WI, USA) following the supplier’s instructions. The voucher specimen (paraype no. Lcy009) was deposited in Taiwan Insect Museum, Taipei, Taiwan and the voucher specimen’s genomic DNA was deposited in the Taiwan Forestry Research Institute, Taipei, Taiwan. The complete mitogenome of *L. chengyuani* was sequenced using the next-generation sequencing method (Illumina MiSeq, San Diego, CA, USA) (Hahn et al. 2013). A total of 12 Gb next-generation sequencing paired-end reads were used to assemble the complete mitogenome sequence. The CLC Genomics Workbench (QIAGEN) was used for sequence quality analysis, data trimming, and de novo assembling. The locations of the protein-coding genes, ribosomal RNAs (rRNAs), and transfer RNAs (tRNAs) were predicted by using MITOS Web Server (Bernt et al. 2013) and identified by alignment with other mitogenomes of Lucanidae. The AT and GC skew were calculated according to the following formulas: AT skew = (A–T)/(A + T) and GC skew = (G–C)/(G + C) (Perna and Kocher 1995). The complete mitogenome of *L. chengyuani* is 16,926 bp in length (GenBank Accession No. MK878514), including 13 protein-coding genes, 2 rRNA genes, 22 tRNA genes, and 1 control region. The nucleotide compositions of the *L. chengyuani* mitogenome were 38.37% for A, 27.96% for T, 23.03% for C, and 10.637% for G. The AT and GC skewness of mitogenome sequence were 0.157 and -0.368 showing the A-skew and C-skew. The gene rearrangement of the mitogenome in *L. chengyuani* is identical to the inferred ancestral insect type (Cameron 2014).

We reconstructed the phylogenetic relationships of Scarabaeoidea including 9 Lucanid species and 5 Scarabaeid species, and *Xylosandrus crassiusculus* (Curculionidae) as outgroup using Mrbayes v. 3.2.4 (Qiagen, Shanghai, China) (Huelsenbeck and Ronquist 2001) under the molecular evolutionary model GTR + I+G (Figure 1). Nodal supports were indicated by posterior probabilities. The clade including *L. chengyuani* and the other eight Lucanid species received absolute support (100%). The clade including *Neolucanus maximus* and *Odontolabis cuvera* is sister to all the other stag beetle clades, which contain *L. chengyuani* and *L. mazama*. Our result of phylogenetic reconstruction is not consistent with the result from a previous study of Kim and Farrell (2015). More complete mitogenomic data from other Lucanid species are needed for further studies on the phylogeny of Lucanidae. Mitogenomic data from this study will provide useful information for further studies for the population genetics, speciation, biogeography, and conservation of *L. chengyuan*i in the future.

**Figure 1.**
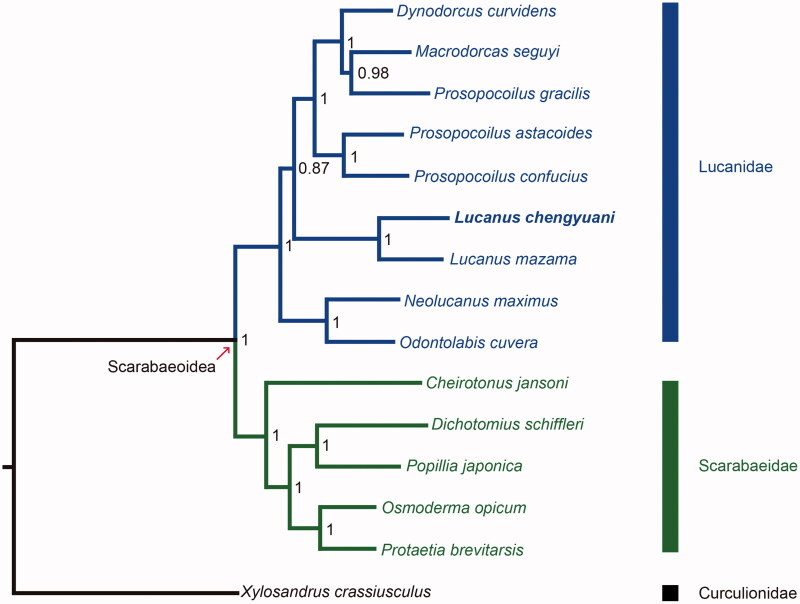
Phylogenetic tree of the 9 Lucanid species, 5 Scarabaeid species, and 1 Curculionid species based on the sequences of 13 mitochondrial protein-coding genes. The phylogenetic tree was inferred with Mrbayes v. 3.2.4 (Huelsenbeck and Ronquist 2001) under model GTR + I + G. Value on nodes indicated posterior probabilities. *Cheirotonus jansoni* (KC428100) (Shao et al. 2014), *Dichotomius schiffleri* (KY100258) (Amorim et al. 2017), *Dynodorcus curvidens* (MF612067) (Chen et al. 2018), *Lucanus chengyuani* (MK878514, in this study), *Lucanus mazama* (FJ613419) (Sheffield et al. 2009), *Macrodorcas seguyi* (MF612068) (Chen et al. 2018), *Neolucanus maximus* (MF401425), *Odontolabis cuvera* (MF908524), *Osmoderma opicum* (KU500641), *Popillia japonica* (MG971231) (Yang et al. 2018), *Prosopocoilus gracilis* (KP735805), *Prosopocoilus astacoides* (KF364622) (Kim et al. 2015), *Prosopocoilus confucius* (KU552119) (Lin et al. 2017), *Protaetia brevitarsis* (KC775706) (Kim et al. 2014), *Xylosandrus crassiusculus* (KX035196).
